# A retrospective comparative study of arthroscopic fixation in acute Rockwood type IV acromioclavicular joint dislocation: single versus double paired Endobutton technique

**DOI:** 10.1186/s12891-018-2104-9

**Published:** 2018-05-24

**Authors:** Jian Xu, Haifeng Liu, Wei Lu, Dingfu Li, Weimin Zhu, Kan Ouyang, Bing Wu, Liangquan Peng, Daping Wang

**Affiliations:** Department of Sports Medicine, Shenzhen Second People’s Hospital, Shenzhen First Affiliated Hospital, Shenzhen University, No.3002 Sungang West Road, Futian district, Shenzhen City, 518000 Guangdong Province China

**Keywords:** Acromioclavicular joint dislocation,·arthroscopic fixation,· Endobutton technique

## Abstract

**Background:**

Rockwood type IV acromioclavicular joint (ACJ) dislocation is a trauma usually needs surgical treatment. Paired EndoButton technique (PET) is used in treating such condition. However, the effect of using different types of PET (single versus double PET) for fixation remains controversial. This study aims to evaluate and compare the efficacy of single and double PET and to provide a suitable option for the surgeons.

**Methods:**

We retrospectively reviewed the charts of patients with acute Rockwood type IV ACJ dislocation who had undergone arthroscopic fixation using single or double PET fixation between March 2009 and March 2015. Seventy-eight consecutive patients identified from chart review were picked and were divided into the single and double PET group with 39 cases in each group. The indexes of visual analog scale score (VAS) for pain, the radiographs of the affected shoulder at different time points of the follow-up, the time of return to activities and sports, the constant functional score, and the Karlsson acromioclavicular joint (ACJ) score, were assessed in a minimum of 2 years postoperation.

**Results:**

The average coracoclavicular (CC) and acromioclavicular (AC) distances of the affected joints in the double PET group were significantly smaller than those of the single PET group 2 years postoperation (*P < 0.05*). The average AC and CC distances in the healthy shoulder joints were significantly smaller than those of the affected joints in the single PET group (*P < 0.05*); however, these values were not significantly different from those of the affected joints in the double PET group (*P > 0.05*). The mean VAS pain score was not significantly different, while significant difference was found for the number and times of cases return to activities and sports, constant functional score, and Karlsson ACJ score (*P < 0.05)* between the two groups. Therefore, the double PET group has better outcome than the single PET group. Complications including redislocation, button slippage, erosion, or AC joint instability occurred in the single PET group, while the complication in the double PET group was rare.

**Conclusions:**

Compared with the single PET, the double PET group achieved better outcome with less complications in arthroscopically treating acute Rockwood type IV ACJ dislocation.

## Background

Treatment of acromioclavicular joint (ACJ) dislocation is usually guided by the Rockwood classification [1, 2], and surgery is typically performed in high grade ACJ dislocation [3]. Rockwood type IV ACJ dislocation needs surgical treatment [4, 5]. However, the suitable surgical methods remain controversial. Surgeons have treated this condition using hook plate [4, 6]. In 2007, Steven Struhl first reported excellent outcome through open surgery using EndoButton device for fixation of ACJ dislocation [7].

Some authors have reported good results by using arthroscopic paired EndoButton technique (PET) for fixation [8–15], arthroscopic visualization allows precise positioning of the EndoButton at the coracoid base, thus keeping the operation far from the nervous structures with respect to the open approach. The arthroscopic technique can diagnose and treat associated lesions.. However, the outcomes were different according to the type of PET fixation; among these types, the single and double PET are mostly used [16–19]. However, the outcome was different according to the different type of PET fixation, of which the single and double PET which are mostly used [5, 12, 20]. To our knowledge, no study has compared the outcomes of single and double PET arthroscopic fixation. We hypothesized that double PET fixation would provide superior results, superior symptom relief, and patient satisfaction.

## Methods

### Patient selection

This retrospective study was approved by the Ethical Committee of the Shenzhen Second People’s Hospital, and all patients gave informed consent before surgeries. We reviewed the charts of patients with acute Rockwood type IV ACJ dislocation and who had undergone arthroscopic fixation using single or double PET fixation between March 2009 and March 2015 in our department. Inclusion criteria were as follows: 1) acute dislocations (< 2 weeks after trauma), 2) age of 18–45 years, 3) Rockwood type IV dislocation diagnosis, 4) absence of osteoporosis, 5) all operations performed by the same group of surgeons, and 6) follow-up time of at least 24 months. Exclusion criteria were as follows: 1) open and old dislocations, 2) previous shoulder complains or surgery, 3) combination with nerve or vascular injury, 4) association with vital organ damage, 5) association with fractures and/or dislocation of other parts of the ipsilateral limb.

Anteroposterior (AP) and lateral scapular (Y) position radiographs of the bilateral shoulder joints and 3D computed tomography (CT) scans of the affected shoulder joints were preoperatively obtained in all cases. All procedures were performed in the beach chair position with the administration of general anesthesia.

### Surgical techniques

All procedures were performed by the same group of surgeons. An anterior cruciate ligament (ACL) tip-to-tip tibial aimer, EndoButton device (Smith & Nephew, MA), and high strength wires (Ultrabraide, Smith & Nephew, MA) were used intraoperatively.

#### Single PET fixation

**Fig. 1:**
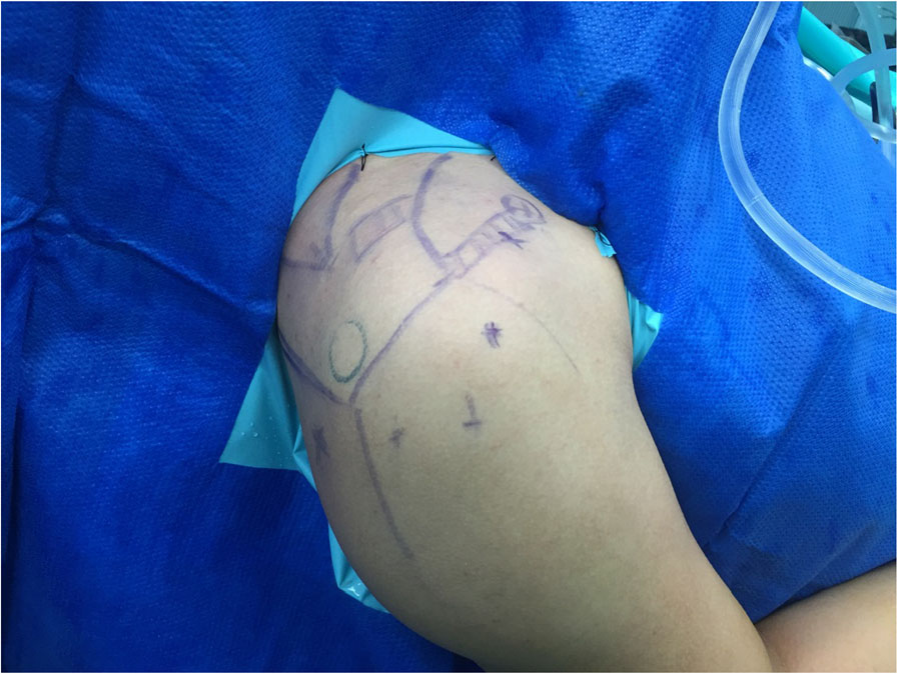
Two portals (standard posterior portal and anterolateral portal) and one 2-cm transverse incision directly over the ACJ were made on the skin

First, a standard posterior portal was established for inspection of the shoulder joint using a 4.0 mm 70° arthroscope. A standard anterolateral portal, which is located on approximately 1 cm of posteriolateral side of the acromion anterolateral portion, was then established (Fig. 1). The anterior capsule was dissected using radiofrequency over the subscapularis tendon. The lower surface of subcoracoid was totally debrided to clearly visualize the coracoid base. Second, a 2 cm transverse incision was made directly over the ACJ. The totally dislocated ACJ was clearly visualized. The distal clavicle was excised 5-8 mm and then the ACJ was reduced, a 2.4 mm Kirschner wire (K-wire) was used to fix it temporarily. Third, the tip of the ACL tibial aimer was positioned at the center of the coracoid base. The targeting tip was positioned at the superior surface of the clavicle 2–3 cm medial to the AC joint line and 5 mm anterior to the rear border of the clavicle (i.e., the dividing point of the rear 1/3 anteroposterior diameter of the clavicle). Then, a 2.4 mm guide pin was drilled from the clavicle down directly in line with the base of the coracoid. Once the subsurface of the coracoid was penetrated, the guide pin was pulled out. A 2–0 polydioxanone suture (PDS), which was used as a guiding suture, was inserted through the tunnel from clavicle to coracoid process with a spinal needle and was pulled out through the anterolateral portal. The original built-in loop on the EndoButton was then removed before deployment. Loaded with three high strength wires with a diameter of 1.5 mm (with six wire limbs), the first EndoButton was placed at the superior surface of the clavicle. The three loaded wires were pulled down through the prepared tunnel from clavicle to coracoid process, then out to the anterolateral portal led by the PDS guiding suture. The second EndoButton loaded with the same wires was pushed in through the anterolateral portal and placed at the lower surface of the coracoid process using a knot pusher. Fluoroscopy was used to ensure that reduction was properly completed and the fixation was in the correct place before removing the temporarily fixed K-wire.

#### Double PET fixation

The steps of shoulder joint inspection, ACJ management, and the first guide pin introduction were the same as previously stated. The only difference was to keep the 2.4 mm guide pin temporarily as a reference after drilling of the first clavicle-coracoid tunnel, and then another 2.4 mm guide pin was drilled from the clavicle into the coracoid by using a guide with two tube, which was paralleled with the first guide pin at 6 mm to 8 mm to the anterolateral side. Furthermore, the second paired loop EndoButton system was placed on the second bone tunnel. Fluoroscopy was also used to ensure that ACJ reduction was completed correctly and the fixation was in secure placement.

### Postoperative rehabilitation

After surgery, a sponge shoulder abduction orthosis was used to fix the shoulder joint at 0 degree external rotation position and was kept for 6 weeks. Forty-eight hours after surgery, all patients were encouraged to participate in the following rehabilitation program: passive abduction, forward flexion, and external rotation with the arm at the pain tolerance level. Active movements for the arm began at 6 weeks postoperation. Normal activities, daily work, and limited rehab sports were allowed at 3 months after surgery.

No difference in the rehabilitation program was noted between the two groups.

### Review of clinical data

Using a retrospective study design, the results were evaluated in a minimum of 2 years after surgical reduction of the ACJ dislocation. The coracoclavicular and acromioclavicular distances measured from the preoperative and 2-year postoperative radiographs in both groups were compared and analyzed. Functional outcomes were assessed including the following aspects: visual analog scale (VAS) for pain (ranging from 0 to 10, with 10 being the worst), radiographs of the affected shoulders and the internal fixation, the range of shoulder motion, the time of return to activities and sports, the constant functional score (ranging from 0 to 100, with 100 being the best), and the Karlsson ACJ score (Grades A–C).

### Statistical analysis

Statistical analyses were performed using SPSS software (version 18.0; SPSS, Chicago, IL). The chi-square and the student t-test were performed differently. All tests were conducted with a 95% confidence interval, in which *P* < .05 was considered statistically significant.

## Results

Out of the 167 reviewed charts for AC joint dislocation patients, 78 fulfilled the above criteria were picked with thirty-nine patients in the single or double PET group. Patients in both groups had similar age, gender, body mass index (BMI), pathological side, and cause of injury (Table 1). Follow-up with a minimum of 2 years was instructed from all patients. Based on the radiographs, the average CC and AC distances of the affected joints were not significantly different between the two groups preoperatively (*P > 0.05*) (Table 2). Postoperatively, the average CC and AC distances measured from the 2-year postoperative radiographs were significantly smaller in both groups (*P < 0.05*) (Table 2). However, the average CC and AC distances in the double PET group were significantly smaller than those of the single PET group 2 years postoperation (*P < 0.05*) (Table 2). Furthermore, the average CC and AC distances in healthy shoulder joints were significantly smaller than those in the affected joints in the single PET group (*P < 0.05*) but were not significantly different from those in the affected joints in the double PET group (*P > 0.05*).

**Table 1:** Baseline characteristics between the two groups

Characteristics	Single PET Group	Double PET Group	*P*
Age (years)	29.4 ± 3.3	31.2 ± 4.5	.363
Sex (male: female, n)	30:9	28:11	.431
The affected side (left: right, n)	13:26	15:24	.422
Cause of injury (road accident: fall, n)	22:17	26:13	.397
body mass index (BMI)	23.4 ± 4.7	25.1 ± 5.3	.788

**Table 2:** Comparison of coracoclavicular and acromioclavicular distance in the single PET and the double PET group measured from preoperative and two-year postoperative radiographs

Group		CC distance(mm)				AC distance(mm)				
	Preoperative(AS)	Postoperative(AS)	Preoperative(HS)	*P* ^*a*^	*P* ^*b*^	Preoperative(AS)	Postoperative(AS)	Preoperative(HS)	*P* ^*a′*^	*P* ^*b′*^
Single PET	13.5 ± 2.8	9.3 ± 1.7	7.8 ± 1.5	<.001	.377	5.7 ± 1.1	2.4 ± 0.8	1.6 ± 0.7	<.001	<.001
Double PET	14.1 ± 3.3	7.6 ± 2.1	7.4 ± 1.9	<.001	0.274	5.4 ± 1.3	1.5 ± 0.9	1.3 ± 0.6	<.001	0.484
*P*	> 0.05	<.001	.766	–	–	.843	<.001	.356		

*AS* affected shoulder, *HS* healthy shoulder; p^a^ and p^a’^ refer to the respective comparison of the coracoclavicular and acromioclavicular distance of the affected shoulder joints measured preoperatively and postoperatively. p^b^ and p^b’^ refer to the respective comparison of the coracoclavicular and acromioclavicular distances of the affected shoulder joints measured postoperatively and the healthy shoulder joints measured preoperatively. *P* refers to the comparison of the single PET and the double PET group

**Fig. 2:**
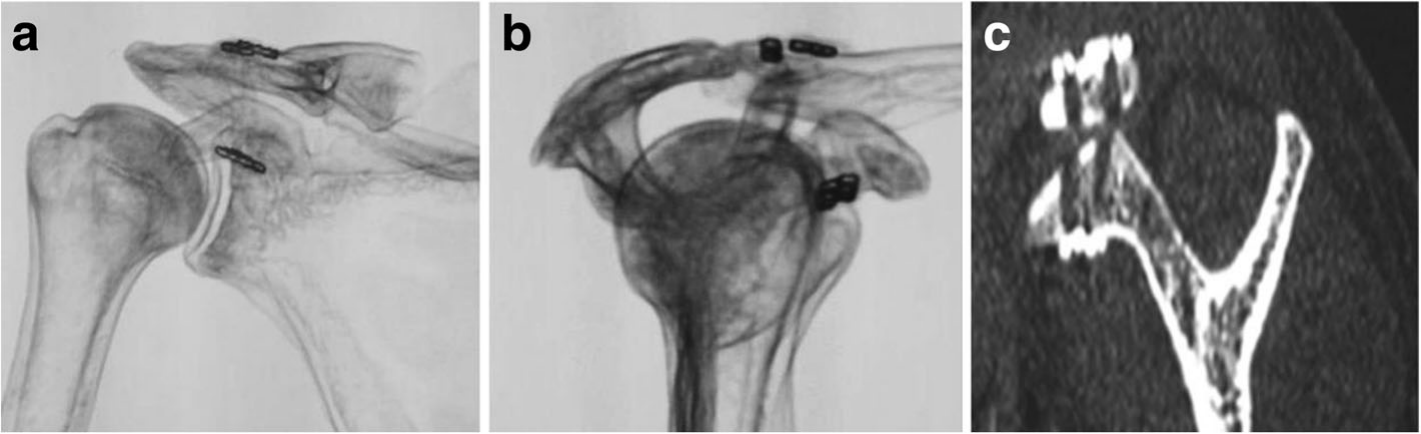
The postoperative imaging showed that the fixation buttons were in proper placement especially in double PET group. (**a**) Shoulder AP view. (**b**) Shoulder “ Y position ” View. (**c**) CT scans view

**Fig. 3:**
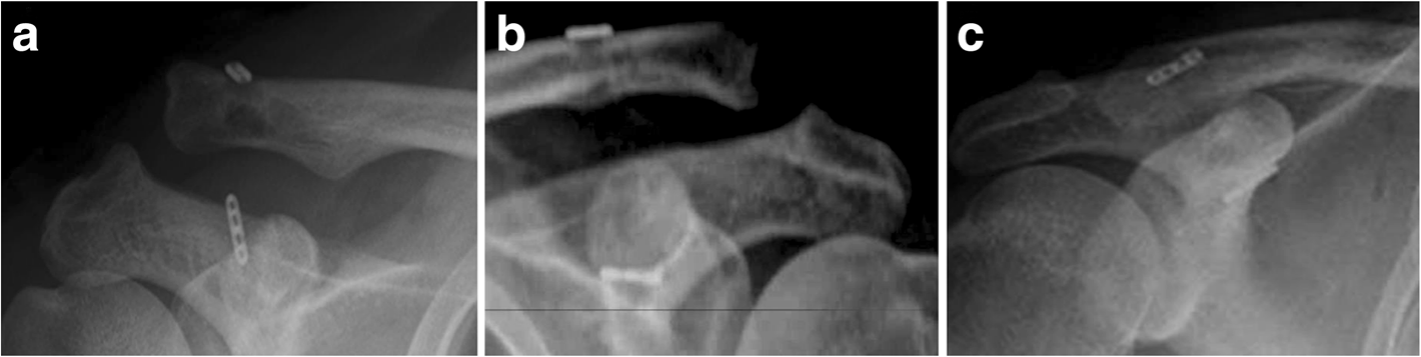
Complications after surgeries in the single PET group: One button on the coracoid side slipped out from the original place in 2 cases in single PLE group (**a**); Rockwood type II AC joint dislocation occurred in four cases: two of them were due to EndoButton separation (**b**), and the other two were due to button eroding into the superior clavicle cortex (**c**)

The EndoButtons were properly placed in the majority of the cases, especially in the double PET group (Fig. 2). However, complications occurred in some cases. In the single PET group, two cases of redislocation were reported because one EndoButton on the coracoid side slipped out from the original place (Fig. 3a). Revision surgeries were successfully performed with previous procedure. One button slipped out twice in one case; thus, a hooked plate was finally used instead. One case acquired infection and was recovered by conservative treatment. Rockwood type II AC joint dislocation occurred in four cases: two of them were due to EndoButton separation (Fig. 3b), and the other two were due to button eroding into the superior clavicle cortex (Fig. 3c). In the double PET group, no button slippage, erosion, or AC joint instability was found. In one case, one pair of buttons was removed due to infection at the clavicle 3 months after surgery; the AC joint remained stable in this case with no dislocation at the final follow-up (Table 3).

**Table 3:** Evaluation Results of Two Fixation techniques (x ± s)

Variables	Preoperation		Postoperation		*p-value*
	Single PET	Double PET	Single PET	Double PET	
Total Complications			7(17.9%)	1(2.5%)	*<.001*
Infection	–	–	1(2.5%)	1(2.5%)	*NS*
Redislocation	–	–	2(5.1%)	0	*<.001*
Loss of reduction	–	–	4(10.3%)	0	*<.001*
Cases return to former sports	–	–	24(61.5%)	35(89.7%)	*<.001*
Time of return to sports (mon)	–	–	5.43 ± 3.33	3.25 ± 2.66	*<.001*
VAS	8.25 ± 0.67	8.00 ± 0.56	1.78 ± 2.22	1.60 ± 1.62	*NS*
Constant Score	23.55 ± 3.36	24.51 ± 1.67	83.2 ± 4.01	92.15 ± 2.88	*<.001*
Karlsson					
A	–	–	26	35	
B	–	–	8	4	*<.001*
C	–	–	5	0	

*PET* Paired Endonbutton Technique

In the single PET group, 15 patients gave up their former sports, and 24 resumed their previous sports activities within an average of 5.3 months (range from 3 to 8 months) postoperatively. In the double PET group, 4 patients abandoned his former sports, and 35 cases resumed their former sports activities in an average of 3.3 months (range from 3 to 4 months) postoperatively. There was a significant difference (*P* < 0.05) between the two groups (Table 3).

After 2 years post operation, the mean VAS pain score was not significantly different for the two groups. However, the mean time to recover shoulder movements, the mean constant functional scores, and the Karlsson ACJ score were significantly different between the two groups, indicating that the double PET group achieved superior results (*P < 0.05*) (Table 3).

## Discussion

Arthroscopic treatment by using PET is valuable in treating acute Rockwood type IV ACJ dislocation [8–11, 21]. PET has the advantage that conforms to the micromotion characteristics of the AC joint. Moreover, the fixation is quite stable and reliable enough to guarantee bone and ligament healing [9, 11, 22]. Spoliti M et al. [10] have found that double TightRope technique can provide shorter distance between the coracoid and clavicle than the single TightRope technique. Walz L [23] used double loop buttons for anatomic fixation of the coracoclavicular ligament in an experimental biomechanical study and confirmed that the PET method led to favorable results. The most important novelty of the present study is its comparison of the double PET with single PET in arthroscopically treating acute ACJ dislocation.

Some complications, including redislocation, button slippage, and AC joint laxity, occurred in the single PET group. These complications may be attributed to the following reasons: 1) The malpositioning of the tunnels 2) The tension of the paired EndoButton bearings was excessive, thus increasing the force of slippage, especially when the EndoButton was laid on the uneven face of the clavicle or the coracoid process. 3) The tension on the clavicle or the coracoid process received from the EndoButton may be too concentrated, causing bone erosion and thus leading to AC joint laxity [8, 19]. 4) The three sutures were perhaps unable to bear such a strong traction force between the coracoid and clavicle, thus resulting in suture lengthening or rupture and causing ACJ laxity or redislocation. Boileau [21] reported the complication of button intraosseous migration using the single PET and concluded that the main reason for such complication is the excessively broad bone tunnel and relatively small button plate area. On the contrary, the results in the double PET group revealed no button slippage, erosion, or AC joint instability. In one case, one pair of buttons was removed due to infection at the clavicle 3 months after surgery, yet no ACJ dislocation or obvious laxity was found. Thus we assume that the ligament can be totally healed in 3 months. The double PET can provide double security for the fixation while dispersing the forces on the bones and EndoButtons. Nevertheless, the six sutures were strong enough to guarantee the stability of the AC joint. Some authors described a fixation technique of acute ACJ dislocation through a Y-shaped bone tunnel configuration for good outcome [10]. This finding indicates that the postoperative complications above can be possibly avoided by using additional wires and EndoButtons. In a cadaveric study [24], Abat found that double bone tunnels can provide better biomechanical properties than a Y-shaped tunnel. Compared with the single PET fixation, the double PET fixation showed that better clinical outcome and allowed quicker rehabilitation and stronger stability.

Despite the merits shown, this study has several limitations. First, we have not measured the stress on both the EndoButtons and the sutures after surgery. This step needs to be improved in further studies. Second, the mean follow-up period is relatively short, especially for those cases that yielded recovery in a short time period. Third, the cases for this study were not as many as we desired. Additional cases must be investigated by using the double PET technique in future clinical work. Moreover, further understanding of the technique is needed to achieve desirable outcomes.

## Conclusions

Compared with the single PET, the double PET achieved better outcome with less complications in arthroscopically treating acute Rockwood type IV ACJ dislocation.
